# The dynamic changes in quantitative parameters of diffusion-weighted imaging at different b-values in a prostate cancer mouse model and their correlation with histopathology parameters

**DOI:** 10.1590/1414-431X2025e14527

**Published:** 2025-08-29

**Authors:** Xin Zhou, Yida Li, Xinyuan Zhang, Mengmeng Zhang, Renfu Zhang, Shengjian Sun, Guohua Li

**Affiliations:** 1 Department of Medical Technology, Qiqihar Medical University, Qiqihar, China; 2 Department of Radiology, The First Affiliated Hospital of Qiqihar Medical University, Qiqihar, China

**Keywords:** Prostate cancer, Diffusion-weighted imaging, Apparent diffusion coefficient, Exponential apparent diffusion coefficient, Tumor nuclear fraction

## Abstract

The aim of this study was to evaluate the dynamic variations in the quantitative parameters of diffusion-weighted imaging (DWI) at different b-value combinations in a prostate cancer (PCa) mouse model for noninvasive monitoring of histopathological changes. Twenty-five male C57BL/6J mice were randomly allocated into a control group (n=5) or an experimental group (n=20). The experimental groups were used to establish the PCa model. On days 9, 12, 15, and 18 post-modeling, 5 mice were randomly selected for MRI, including T1WI, T2WI, T2WI SPIR, and DWI. The b-values were set at 0, 500, 1000, 1500, and 2000 s/mm^2^. Apparent diffusion coefficient (ADC) and exponential apparent diffusion coefficient (EADC) values from different b-value combinations were measured. Post-MRI, tumors were excised for histopathological analysis. DWI quantitative parameters, tumor nuclear fraction, and Ki-67 area fraction were compared on different days, along with correlation analysis. ADC values gradually decreased as tumor progressed, whereas EADC values gradually increased. Tumor nuclear fraction increased over time. Ki-67 increased first and then decreased. Tumor nuclear fraction was negatively correlated with the ADC value and positively correlated with the EADC value. The Ki-67 was positively correlated with the ADC value and negatively correlated with the EADC value. ADC values at b=1000, 1500 s/mm^2^ and the EADC values at b=0, 500 s/mm^2^ demonstrated the strongest correlations with the tumor nuclear fraction; the ADC and EADC values at b=500, 1000 s/mm^2^ were more strongly correlated with Ki-67, being potential noninvasive imaging biomarkers for monitoring changes in tumor histopathology.

## Introduction

Prostate cancer (PCa) represents one of the most prevalent malignant tumors in males globally, ranking second to lung cancer in terms of mortality (1,2). PCa is characterized by rapid progression and marked heterogeneity (3,4). Accurately estimating tumor proliferation conditions is crucial for grading tissue, tailoring treatments to individual patients, and evaluating treatment efficacy (5,6). However, the procedure for obtaining human pathological biopsies is invasive (7,8), complicating the dynamic observation of histopathological alterations in tumors. Consequently, there is a critical demand for noninvasive methodologies capable of monitoring histopathological alterations, facilitating continuous observation and supporting clinical interventions.

Magnetic resonance imaging (MRI) is a noninvasive approach with superior soft tissue resolution. Multiparametric magnetic resonance imaging (mp-MRI) is extensively applied to diagnose, stage, and monitor prostate cancer (9-11). Diffusion-weighted imaging (DWI), a functional imaging sequence, allows for the routine calculation of apparent diffusion coefficient (ADC) values to assess the diffusion of water molecules in tissue (12,13). These quantitative parameters will effectively evaluate histopathological and biological changes (14-16). However, previous studies used only a fixed combination of b-values in DWI for quantitative parameter determination (17,18) and did not explore the significance of the optimal diffusion sensitivity factor recommended by the latest Prostate Imaging Reporting and Data System (PI-RADS v2.1) or the combination of multi b-values (19). In addition, no study has investigated the relationship between the exponential apparent diffusion coefficient (EADC) and the pathologic progression of prostate cancer.

In this study, we established a C57BL/6J mouse model of prostate cancer and conducted multi b-value DWI at various stages of tumor progression. The objective of this study was to identify which combination of b-values is more effective for noninvasive and dynamic monitoring of tumor growth and pathological changes, thereby providing valuable insights for preclinical research, active surveillance, and assessment of therapeutic interventions in prostate cancer.

## Material and Methods

### Experimental animals

The Animal Ethical Care Committee of Qiqihar Medical University approved the animal experimental protocol. In this study, we selected 25 C57BL/6J male mice, aged 6-8 weeks and weighing 18-22 g, sourced from Liaoning Changsheng Biotechnology Co., Ltd. [License No. SCXK (Liao) 2020-0001; China]. The animal facility maintained regular ventilation with constant temperature and humidity (temperature: 20-24°C, humidity: 45-55%). The mice had *ad libitum* access to food and water. The mice were randomly allocated to the control group (n=5) or the experimental groups (n=20) via a random number table. Within the experimental group, five mice were selected randomly for further experiments on the 9th, 12th, 15th, and 18th days post-modeling.

### Preparation of the orthotopic PCa animal model

The mouse prostate cancer cell line RM-1 was cultured in RPMI-1640 medium supplemented with 10% fetal bovine serum and 1% penicillin-streptomycin solution (100 U/mL penicillin and 0.1 mg/mL streptomycin) and incubated in a 5% CO_2_ atmosphere at 37°C. The cells in the logarithmic growth phase were harvested, digested, and resuspended at a concentration of 2×10^7^ cells/mL. The mice were anesthetized with 4% pentobarbital sodium and immobilized in a supine position on cardboard. The abdominal skin was disinfected before surgery. Under a stereomicroscope, a transverse incision approximately 1.0 cm in length was made in the midline position at the base of the thigh, adjacent to both sides of the lower abdomen, to access the abdominal cavity. The bladder was located and repositioned cranially via tweezers, displacing surrounding adipose tissue to reveal the prostate. A microinjector introduced 10 μL of the RM-1 cell suspension into the prostate capsule, with a visible bulge indicating successful injection. The organs were repositioned, and the abdominal muscle and skin were sutured separately via 6-0 monofilament sutures. Post-operation, the wounds were disinfected with iodophor. The mice in the control group received standard care.

### MRI techniques

All the scans were performed via a 3.0 T MR scanner (Achieva; Philips Healthcare, The Netherlands) with an 8-channel knee coil. Within the experimental group, five mice were randomly chosen for data collection and underwent conventional MRI and multi b-value DWI sequences on the 9th, 12th, 15th, and 18th days post-modeling. Five control mice were subjected to repeated scanning. Post-anesthesia, mice were positioned prone on a custom water model for scanning. The sequence and parameter details were as follows: coronal fast spin echo (FSE)-T1-weighted imaging (T1WI): repetition time (TR) 633 ms, echo time (TE) 10 ms, field of view (FOV) 100×100 mm, matrix 192×155, slice thickness 2 mm, slice gap 0.2 mm, number of excitations (NEX) 2; coronal FSE-T2-weighted imaging (T2WI): TR 3000 ms, TE 80 ms, FOV 100×100 mm, matrix 192×155, slice thickness 2 mm, slice gap 0.2 mm, NEX 2; axial T1WI, T2WI, T2WI spectral pre-saturation with inversion recovery (SPIR): matrix 212×161, with other parameters consistent with the aforementioned settings; and coronal echo planar imaging (EPI)-DWI: TR 2000 ms, TE 67 ms, FOV 120×120 mm, matrix 64×63, slice thickness 2 mm, slice gap 0.2 mm, NEX 5, and diffusion sensitivity factor (b-values) set at 0, 500, 1000, 1500, and 2000 s/mm^2^.

### MRI post-processing and quantitative measurements

The Philips IntelliSpace Portal post-processing workstation facilitated image processing, generating ADC and EADC maps via MR Diffusion software (Achieva; Philips Healthcare). The delineation of the region of interest (ROI) and measurement of pertinent parameters were collaboratively conducted by two experienced radiologists, with consultations from senior radiologists sought in instances of differing opinions. Both were blinded to the grouping of the mice and the histopathological results. The methodology for calculating the volume of MRI-measured tumors involved measuring the maximum anterior-posterior diameter (a), maximum transverse diameter (b), and maximum longitudinal diameter (c) of the prostate tumor via coronal and axial T2WI. The tumor volume was computed via the following formula: V = a × b × c × 0.52 mm^3^. T2WI served as the anatomical reference for ROI delineation. On DWI with a b-value of 0 s/mm^2^, the largest layer of the tumor was chosen, and three circular ROIs of 3 mm^2^ each were positioned to circumvent areas of hemorrhage and necrosis. ADC and EADC reconstructions were conducted at b-values combinations of 500, 1000, 1500, 2000 s/mm^2^ and 0 s/mm^2^, respectively, with the average ADC and EADC values of the ROIs automatically calculated. By modifying the combinations of b-values (b=500, 1000/1500/2000 s/mm^2^, b=1000, 1500/2000 s/mm^2^, and b=1500, 2000 s/mm^2^), the ADC and EADC values of different combinations of b-values were obtained. The quantitative parameters were derived from the monoexponential equation (20):

$$ADC=\frac{\ln\left(\frac{S_{1}}{S_{2}}\right)}{b_{2}-b_{1}},$$ (Eq. 1)

where S denotes the signal intensity at different b-values, b denotes the b-value, and *EADC* = *S*_2_ / *S*_1_. The three ROIs delineated on DWI were transferred to the coronal T2WI, and three ROIs of equivalent size were delineated on the right thigh muscle within the same plane. The signal intensity ratio between them was designated by the signal intensity ratio (SIR) of prostate cancer, which signifies the T2WI signal of the tumor. The measurement formula is as follows: *SIR* = *S*_*tumor*_ / *S*_*muscle*_.

### Histopathological analysis

Following MRI examination, tumor tissues from the mice in the experimental group were excised after cardiac perfusion with 4% paraformaldehyde. Normal prostate tissue from the mice in the control group was excised in the same way after an MRI examination on the 18th day. These tissues were subsequently rinsed with saline. Three-dimensional diameters were measured via calipers to determine the tumor volume. The tissues were then fixed in 4% paraformaldehyde for 48 h, dehydrated, and embedded in paraffin. Paraffin-embedded sections, approximately 4-µm thick, were subjected to hematoxylin and eosin (HE) staining to facilitate observation of viable tissue, necrosis, and tumor invasion in prostate samples under a microscope (Nikon, Japan). In the experimental group, three sections per mouse sample were chosen at random, and within each section, three fields were selected for analysis at 400× magnification to compute the mean tumor nuclear fraction. The methodology for assessing the tumor nuclear fraction (6,21) involved the use of ImageJ software (v.1.53; National Institutes of Health, USA) to convert HE-stained images to 8-bit grayscale images, setting the threshold from 0 to 110 to isolate the nuclear area. The tumor nuclear fraction was calculated as the ratio of the nuclear area to the total field area, reported as a percentage.

### Ki-67 immunohistochemical staining

The paraffin-embedded sections were dewaxed, hydrated, heated at high temperature for 10 min, inactivated for 10 min, and the serum was blocked for 1 h. A 1:400 dilution of rabbit anti-Ki-67 polyclonal antibody (Bioss, China) was incubated at 4°C overnight, the goat anti-rabbit antibody was incubated at room temperature for 1 h, streptavidin was added at room temperature for 30 min, color was developed, restaining was performed, and, after differentiation, the water was washed back to blue, and the tablets were sealed. A phosphoric acid buffer salt solution was used as a negative control, a normal spleen was used as a positive control, and brown staining of the cell nucleus was used as a positive control. Three sections were selected for each sample in the experimental group, and three fields of view were randomly selected for each section under 400× magnification. Positive areas were extracted via color deconvolution via ImageJ software, and the area fraction of the three fields was measured to calculate the average value, which was used to characterize the proliferation ability of the tumor cells.

### Statistical analysis

Data analysis was conducted via SPSS software (v.26.0; IBM, USA). All the data are reported as means±SD. The Shapiro-Wilk test was used to assess the normality of the data. Unpaired *t*-tests were used for two-group comparisons on normally distributed data, and comparisons across multiple groups were performed via one-way analysis of variance (ANOVA). Two-group comparisons were performed with the Mann-Whitney *U* test on non-normally distributed data, whereas the Kruskal-Wallis test was employed for multiple-group comparisons. Spearman's rank correlation coefficient was used for correlation analysis. A P-value of less than 0.05 was considered statistically significant.

## Results

### General situations

**Figure 1:**
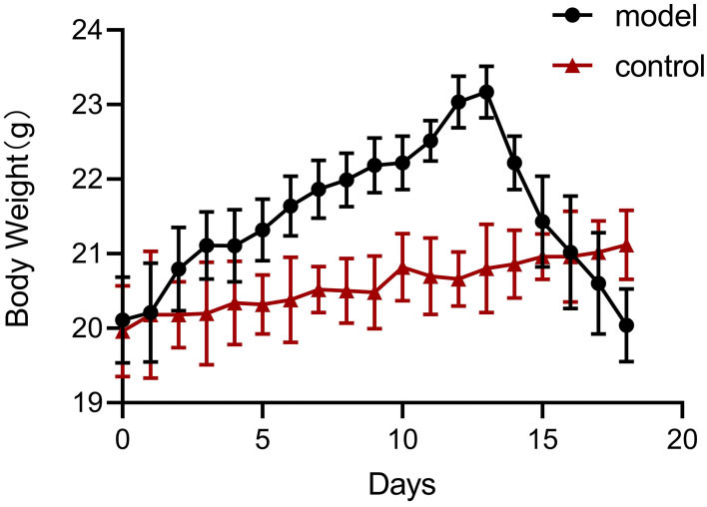
Changes in the body weight of mice in the experimental and control groups after modeling. Data are reported as means and SD.

All the mice survived until the conclusion of their respective experiments. Initially, the experimental group exhibited satisfactory health conditions, characterized by smooth fur and normal eating behaviors, mirroring those observed in the control group. However, beginning on day 12 post-modeling, a noticeable decline in the health status of the experimental mice was observed. This decline was marked by the thinning and coarsening of their fur, a reduced frequency of eating, drinking, and defecation, and a significant decrease in activity levels. Palpation revealed a hard mass in the lower abdomen. The body weights in the experimental group initially increased post-modeling but significantly decreased in the later stages. The weights of the mice in the control group showed a steady upward trend, without a sharp upward or downward trend (Figure 1).

### MRI results

**Figure 2:**
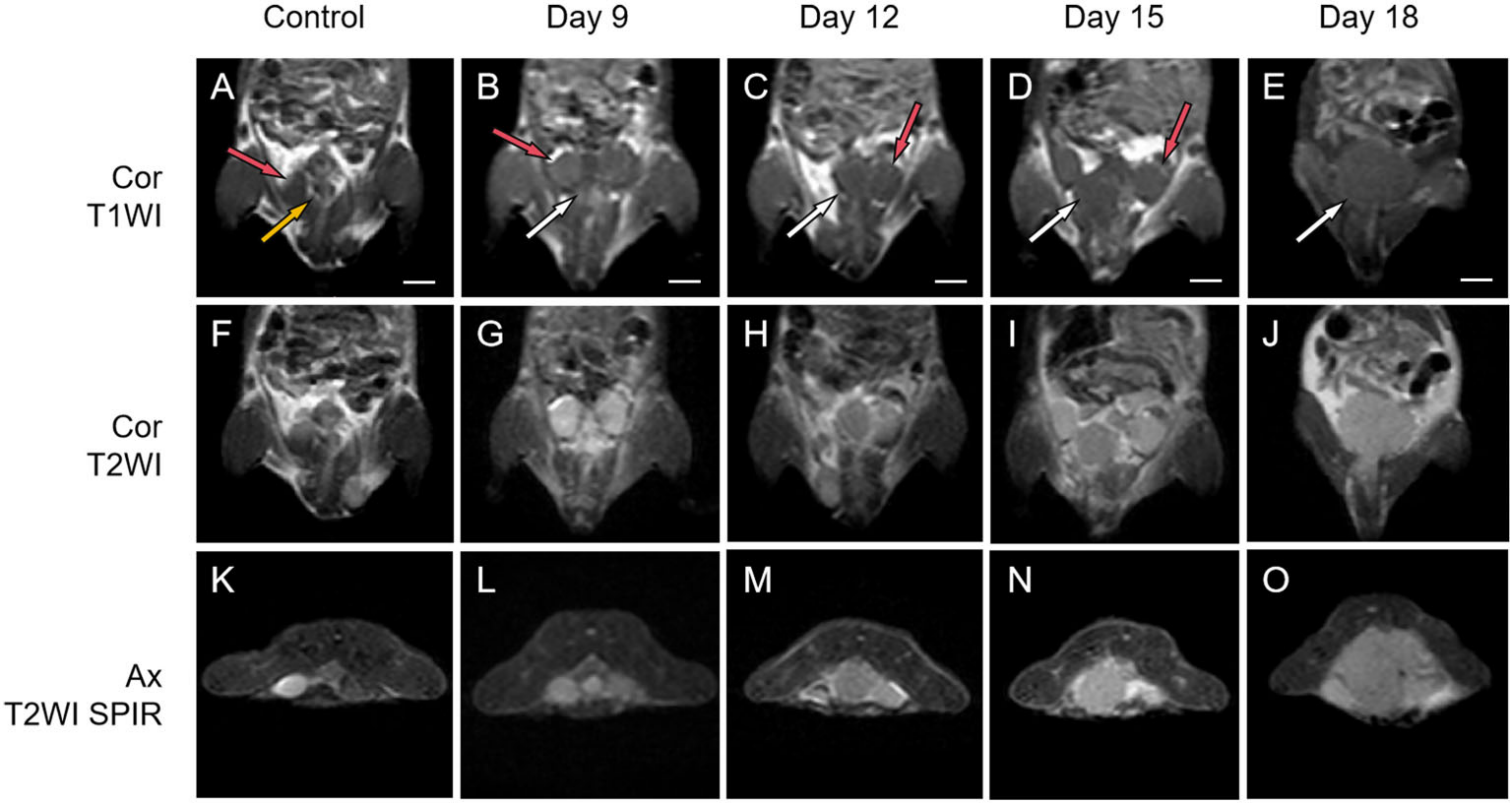
Standard MRI sequences of the mice. **A**-**E**, Coronal T1WI images of mice from the control and experimental groups. **F-J**, Coronal T2WI images from each group. **K-O**, Axial T2WI SPIR from each group. Red arrows indicate testicles, yellow arrows denote normal prostate tissue, and white arrows point to prostate tumors. Prostate tumors in the experimental group were discernible across all MRI sequences, with clear delineation from adjacent tissues and a noted increase in size over the observation period. MRI: magnetic resonance imaging; T1WI: T1-weighted imaging; T2WI: T2-weighted imaging; T2WI SPIR: T2-weighted imaging with spectral pre-saturation with inversion recovery. Scale bar 500 μm.

MRI of the mice in the experimental group at different time points revealed tumor formation in the lower abdomen (Figure 2), which was challenging to differentiate from the normal prostate. The prostate tumor displayed a low signal on T1WI, closely resembling the signal of the testes, and a medium to high signal on T2WI and T2WI SPIR, slightly lower than that of the testes, with a well-defined margin and homogeneous signal. In cross-sectional views, the tumor was confined within the pelvic cavity. The tumor size progressively increased, with scattered areas of long T1 and long T2 necrotic signals emerging within the tumor on days 15 and 18. By day 18, the tumor had expanded to fill the lower abdomen and exert pressure on adjacent tissues, occupying the pelvic cavity in cross-sectional views. In contrast, the prostates of the control group mice, which were situated below the bladder, presented medium to low signals on both T1WI and T2WI, with well-defined margins. No significant difference was found in the images on the different days.

**Figure 3:**
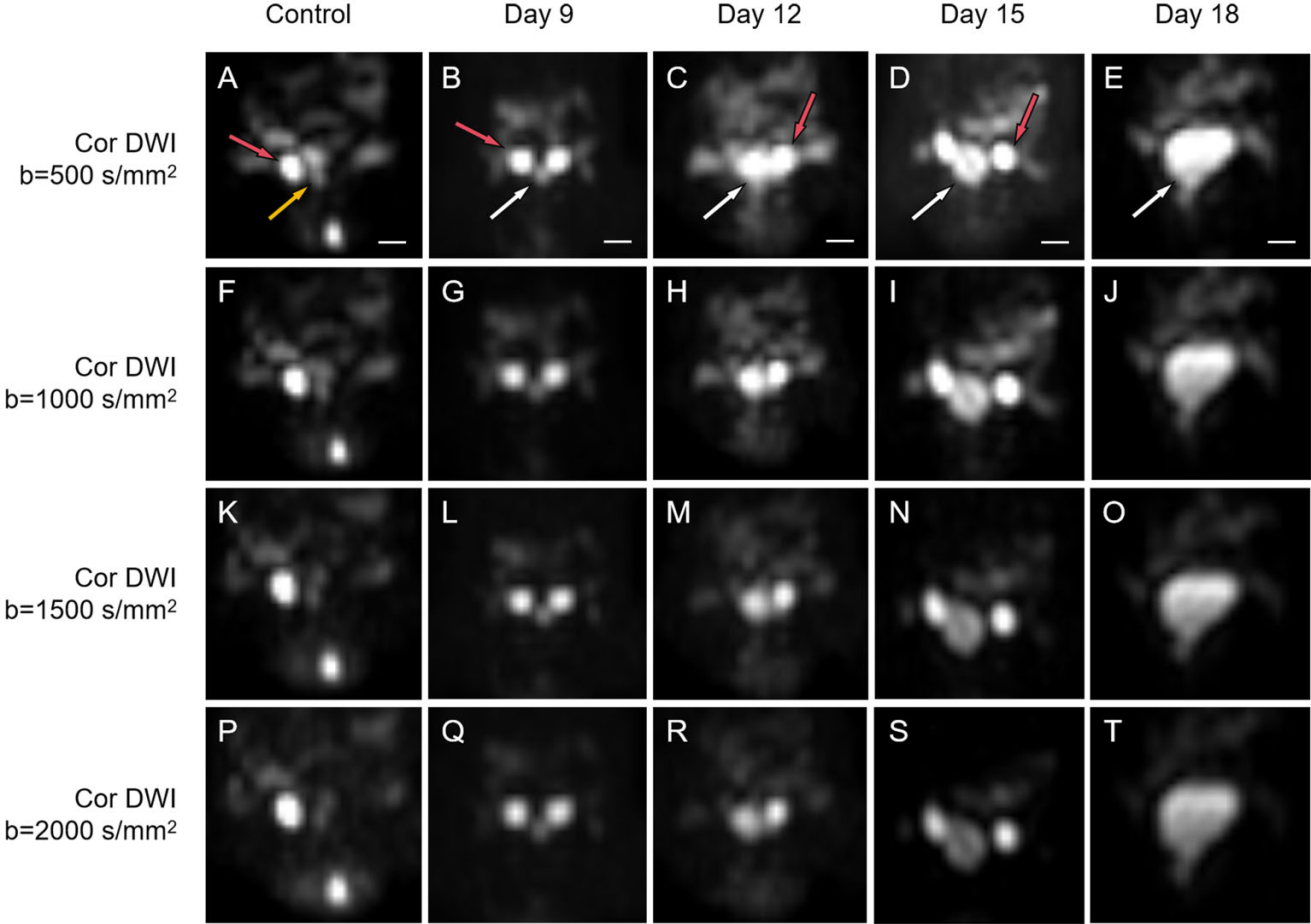
Diffusion-weighted imaging (DWI) of mice at various b-values. **A**-**E**, Coronal DWI of mice from the control and experimental groups at a b-value of 500 s/mm^2^. **F-J**, Coronal DWI at a b-value of 1000 s/mm^2^ for each group. **K-O**, Coronal DWI at a b-value of 1500 s/mm^2^ for each group. **P-T**, Coronal DWI at a b-value of 2000 s/mm^2^ for each group. Red arrows point to testicles, yellow arrows indicate normal prostate tissue, and white arrows indicate prostate tumors. DWI revealed that prostate tumors in the experimental group presented high signal intensity, which became more pronounced over time. As the b-values increase, the signal intensity of prostate tumors decreases at the same time points. Conversely, the DWI signals of normal prostate tissue in the control group were significantly lower than the testicles and prostate tumors. Scale bar 500 μm.

The tumor exhibited a markedly high signal intensity on DWI (Figure 3), marginally lower than the testes. Over time, the tumor's signal intensity increased, creating a stark contrast with the adjacent tissue. As the b-values increased, the tumor signal intensity progressively decreased, yet it remained substantially greater than the surrounding tissue. In the control group, the mouse prostate displayed a slightly elevated signal on DWI.

MRI volume measurements revealed a progressive increase in tumor size over time (Table 1), with the volumetric differences between groups reaching statistical significance (P<0.05).

**Table 1:** Comparison of tumor volume and signal intensity ratio (SIR) of prostate cancer at each time in the experimental group (n=5).

Group	Tumor volume of MRI (mm^3^)	Tumor volume of autopsy (mm^3^)	SIR
Day 9	43.55±13.42^d^	46.84±12.06^d^	2.76±0.61^a^
Day 12	186.55±44.34^c^	187.23±48.51^c^	2.61±0.26^a^
Day 15	634.26±96.91^b^	663.57±100.72^b^	2.51±0.42^a^
Day 18	1076.56±53.77^a^	1110.1±51.19^a^	2.53±0.33^a^
F	303.2	306.7	1.039
P	<0.001	<0.001	0.3825

Data are reported as means and SD. Different letters indicate statistically significant differences (P<0.05; ANOVA). MRI: magnetic resonance imaging. F: statistical results of data comparison among multiple groups.

### Changes in the ADC and EADC values

**Figure 4:**
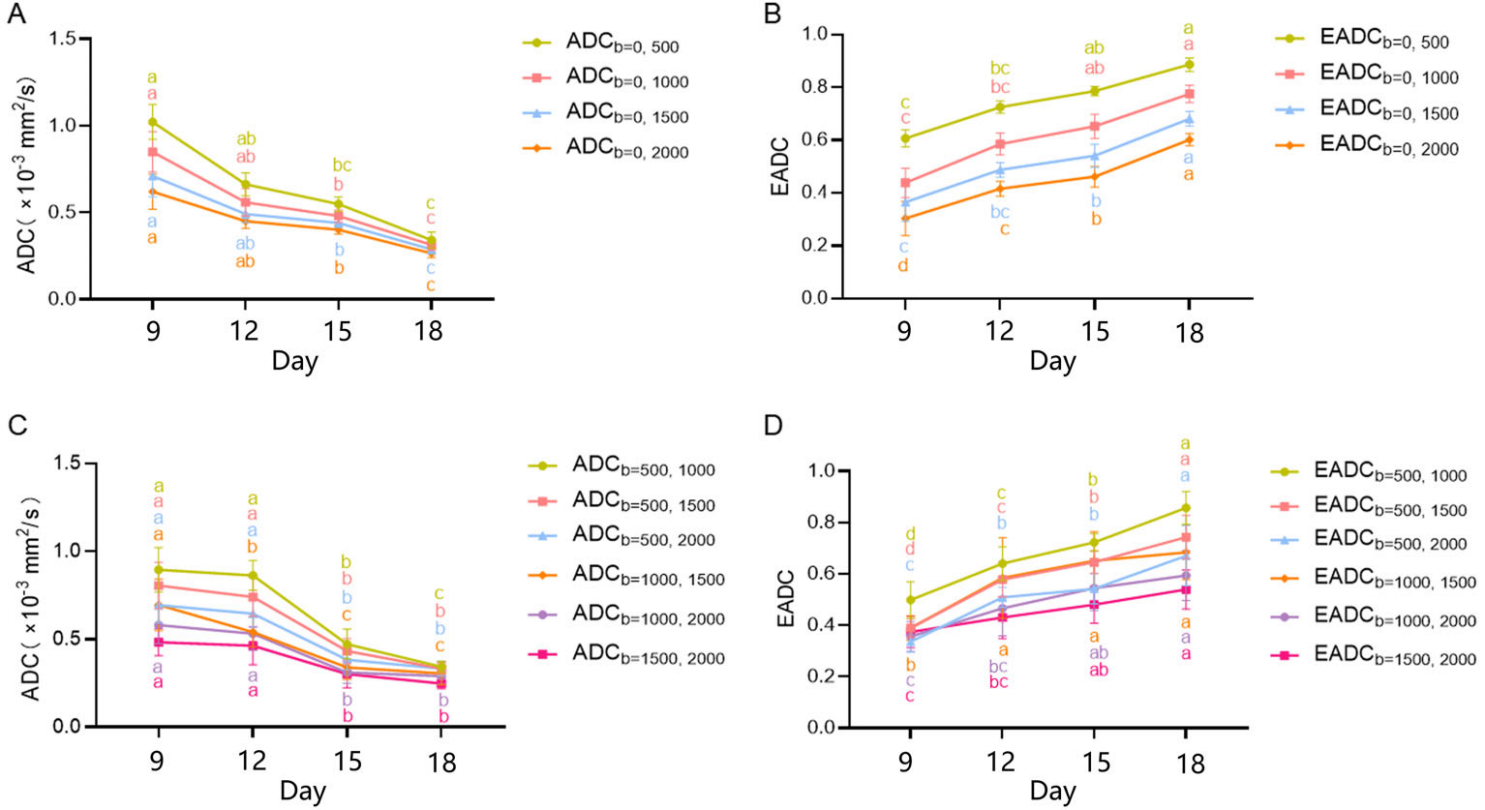
Temporal evolution of quantitative DWI parameters in mice at days 9, 12, 15, and 18. **A**, ADC value variation across different b-value combinations (b=0, 500/1000/1500/2000 s/mm^2^) in the experimental group. **B**, EADC value variation across different b-value combinations (b=0, 500/1000/1500/2000 s/mm^2^) in the experimental group. **C**, Variations in the ADC values across different b-value combinations [(b=500, 1000/1500/2000 s/mm^2^), (b=1000, 1500/2000 s/mm^2^), (b=1500, 2000 s/mm^2^)] in the experimental group. **D**, Variation in EADC values across different b-value combinations [(b=500, 1000/1500/2000 s/mm^2^), (b=1000, 1500/2000 s/mm^2^), (b=1500, 2000 s/mm^2^)] in the experimental group. The ADC values of each b-value combination gradually decreased with time, whereas the EADC values of each b-value combination gradually increased. Data are reported as means and SD. Different letters indicate statistically significant differences (P<0.05) (ANOVA and Kruskal-Wallis test). DWI: diffusion-weighted imaging; ADC: apparent diffusion coefficient; EADC: exponential apparent diffusion coefficient.

The ADC values at b-value combinations (b=0, 500/1000/1500/2000 s/mm^2^) for the experimental group at various time points were consistently lower than those of the control group (P<0.001, Table 2), indicating a decreasing trend over time (Figure 4).

**Table 2:** Comparison of apparent diffusion coefficient (ADC) values from b-value combinations (b=0, 500/1000/1500/2000 s/mm^2^) in each group (n=5) (×10^-3^ mm^2^/s).

Group	b=0, 500 s/mm^2^		b=0, 1000 s/mm^2^		b=0, 1500 s/mm^2^		b=0, 2000 s/mm^2^	
	Model	Control	Model	Control	Model	Control	Model	Control
Day 9	1.02±0.1^a^	1.26±0.04	0.85±0.12^a^	1.15±0.03	0.71±0.12^a^	0.96±0.03	0.62±0.1^a^	0.88±0.02
Day 12	0.66±0.07^ab^	1.26±0.05	0.56±0.08^ab^	1.16±0.03	0.49±0.05^ab^	0.95±0.03	0.45±0.04^ab^	0.87±0.03
Day 15	0.55±0.04^bc^	1.25±0.04	0.48±0.04^b^	1.17±0.03	0.44 ±0.03^b^	0.95±0.03	0.4±0.02^b^	0.88±0.03
Day 18	0.34±0.05^c^	1.25±0.04	0.31±0.03^c^	1.16±0.02	0.29±0.03^c^	0.95±0.03	0.27±0.02^c^	0.86±0.03
F/H	54.05	0.2897	51.93	1.075	52.46	1.549	52.39	1.414
P	<0.001	0.8327	<0.001	0.3672	<0.001	0.671	<0.001	0.7022

Data are reported as means and SD. Different letters indicate statistically significant differences (P<0.05; ANOVA and Kruskal-Wallis test). F/H: statistical results of data comparison among multiple groups.

In the experimental group, the ADC values of the remaining b-value combinations (b=500, 1000/1500/2000 s/mm^2^; b=1000, 1500/2000 s/mm^2^; and b=1500, 2000 s/mm^2^) also exhibited a decreasing trend over time (P<0.001, Supplementary Table S1, Figure 4). Except the ADC values at b=1500 and 2000 s/mm^2^, there were no significant differences between the experimental group and the control group at the other b-value combinations on the 9th day (P>0.05). On the 12th day, there were no significant differences in the ADC values between the experimental and control groups when b=500, 1000/2000 s/mm^2^ (P>0.05). Moreover, statistically significant differences emerged between the experimental and control groups at other time points (P<0.001).

No significant differences were observed in the ADC values at different b-value combinations for the control group at various time points (P>0.05, Table 2 and Supplementary Table S1).

The EADC values at b-value combinations (b=0, 500/1000/1500/2000 s/mm^2^) for the experimental group at various time points tended to increase over time (Figure 4) and were consistently greater than those of the control group (P<0.001, Table 3).

**Table 3:** Comparison of exponential apparent diffusion coefficient (EADC) values from b-value combinations (b=0, 500/1000/1500/2000 s/mm^2^) in each group (n=5).

Group	b=0, 500 s/mm^2^		b=0, 1000 s/mm^2^		b=0, 1500 s/mm^2^		b=0, 2000 s/mm^2^	
	Model	Control	Model	Control	Model	Control	Model	Control
Day 9	0.61±0.03^c^	0.54±0.02	0.44±0.06^c^	0.33±0.01	0.37±0.07^c^	0.23±0.01	0.3±0.06^d^	0.15±0.03
Day 12	0.73±0.02^bc^	0.55±0.03	0.59±0.04^bc^	0.33±0.03	0.49±0.03^bc^	0.23±0.01	0.42±0.03^c^	0.15±0.03
Day 15	0.79±0.02^ab^	0.55±0.03	0.65±0.05^ab^	0.32±0.02	0.54±0.04^b^	0.23±0.02	0.46±0.04^b^	0.17±0.02
Day 18	0.89±0.03^a^	0.54±0.03	0.78±0.03^a^	0.33±0.02	0.68±0.03^a^	0.23±0.02	0.60±0.02^a^	0.16±0.03
F/H	55.39	0.7978	52.81	1.092	50.75	0.3483	131.0	2.132
P	<0.001	0.5003	<0.001	0.3602	<0.001	0.7905	<0.001	0.5454

Data are reported as means and SD. Different letters indicate statistically significant differences (P<0.05; ANOVA and Kruskal-Wallis test). F/H: statistical results of data comparison among multiple groups.

The EADC values of the other b-value combinations (b=500, 1000/1500/2000 s/mm^2^; b=1000, 1500/2000 s/mm^2^; and b=1500, 2000 s/mm^2^) in the experimental group also exhibited a consistent increasing trend over time (P<0.001, Supplementary Table S2, Figure 4). Specifically, at b=1500 and 2000 s/mm^2^, there was no significant difference in the EADC values between the control group and the experimental group on the 9th day (P>0.05); however, statistically significant differences were observed at other time points (P<0.01).

There were no significant differences in the EADC values at different b-value combinations for the control group across various time points (P>0.05, Table 3, Supplementary Table S2).

### Changes in the tumor T2WI signal

In the experimental group, there was no statistically significant difference in the signal intensity ratio of prostate cancer at any time point (P>0.05, Table 1).

### Histopathological analysis and changes in tumor volume

**Figure 5:**
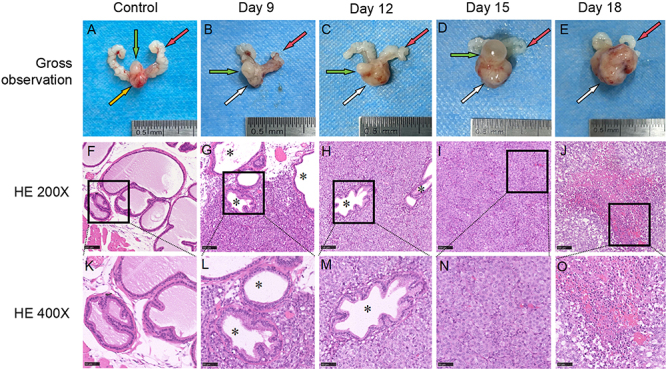
Comparative anatomical and HE-stained images of prostate tumors in mice. **A-E**, General anatomical observation of prostate and prostate tumors from the control and experimental groups. The red arrow indicates the seminal vesicle, the green arrow indicates the bladder, the yellow arrow indicates the normal prostate, and the white arrow indicates the prostate tumor. **F-J**, HE staining of prostate and prostate tumors from the control and experimental groups (×200, scale bar 100 µm). **K**-**O**, HE staining of prostate and prostate tumors from the control and experimental groups (×400, scale bar 50 µm). *Residual normal prostate gland in the tumor; the general anatomy shows that the tumor volume gradually increased with time. HE staining revealed that the tumor cells continued to invade the normal prostate gland, and hemorrhage, necrosis, and structural disorders occurred in the center of the tumor on day 18. HE: hematoxylin and eosin.

Upon dissection of the mice in the experimental group, the tumors below the bladder were identified as irregular solid masses. These masses were connected posteriorly to the seminal vesicles and encapsulated, featuring a tough texture (Figure 5). Partial hemorrhage was observed within the capsule. In the central section of the tumor, the tissue appeared gray-white and interspersed with areas of hemorrhage and necrosis, and distinguishing the residual prostate from the tumor tissue was difficult. Anatomical measurements of the tumor volume revealed a progressive increase over time (Table 1), with statistically significant differences in volume at each time point (P<0.05).

HE staining revealed that the tumor cells were densely packed, exhibited irregular shapes, with visible nucleoli and pronounced nuclear staining and pleomorphism. This cellular arrangement was associated with neutrophil infiltration and abnormal mitotic figures (Figure 5). On the 9th day, HE staining revealed the presence of residual normal prostate glands within the tumor cells, indicating the destruction of basal cells and invasion into the surrounding normal prostate tissue. By the 12th day, a significant reduction in the number of normal prostate glands within the tumor area was observed, with tumor cells continuing to invade surrounding glands and expand their invasion area. On the 15th day, the glandular structure within the tumor area was completely obliterated, the central areas of the tumor displayed scattered hemorrhages, and vacuolation began at the tumor periphery. By the 18th day, lamellar hemorrhage and necrosis were evident in the central area of the tumor, disrupting the internal structure of the tumor and leading to vacuolation and hyalinization.

### Changes in the tumor nuclear fraction

**Figure 6:**
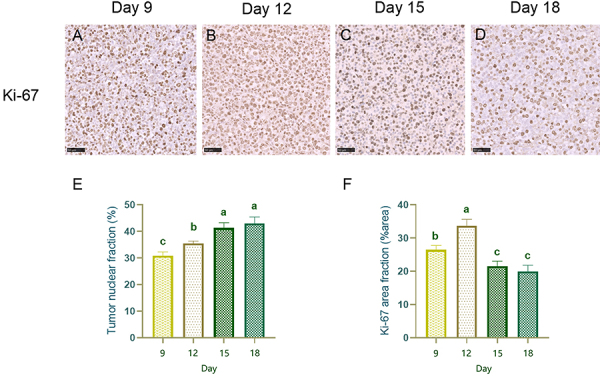
Ki-67 immunohistochemical staining and quantitative analysis of the experimental group. **A**-**D**, Ki-67 immunohistochemical staining of prostate tumors from the experimental group (×400, scale bar 50 µm). Comparative analysis of the tumor nuclear fraction (**E**) and the Ki-67 area fraction (**F**) at days 9, 12, 15, and 18. Ki-67-positive expression was obvious in the tumor cell nucleus and was most intense on day 12. Data are reported as means and SD. Different letters indicate a statistically significant difference (P<0.05; ANOVA).

In the experimental group, the tumor nuclear fraction increased in a time-dependent manner (Figure 6, Table 4). This increase was not statistically significant between the 15th and 18th days (P>0.05). However, significant differences were observed at all other measured time points (P<0.001).

**Table 4:** Comparison of tumor nuclear fraction and Ki-67 area fraction at each time point in the experimental group (n=5).

Group	Tumor nuclear fraction (%)	Ki-67 area fraction (% area)
Day 9	30.94±1.36^c^	26.48±1.31^b^
Day 12	35.53±0.87^b^	33.73±1.92^a^
Day 15	41.34±1.96^a^	21.55±1.51^c^
Day 18	43.03±2.45^a^	20.00±1.86^c^
F	147.9	205.6
P	<0.001	<0.001

Data are reported as means and SD. Different letters indicate statistically significant differences (P<0.05; ANOVA). F: statistical results of data comparison among multiple groups.

### Changes in the Ki-67 area fraction

Ki-67-positive immunohistochemical staining revealed the presence of Ki-67-positive prostate tumor cell nuclei in the experimental group; the tumor cell nuclei were obviously stained at all time points, and the positive nuclei were the densest on the 12th day (Figure 6). The results of the quantitative analysis revealed that the Ki-67 area fraction gradually increased from days 9 to 12 and then began to decrease beginning on day 15 (Figure 6). There was no statistically significant difference between days 15 and 18 (P>0.05), while there was a statistically significant difference between the other time points (P<0.001) (Table 4).

### Correlation analysis

**Figure 7:**
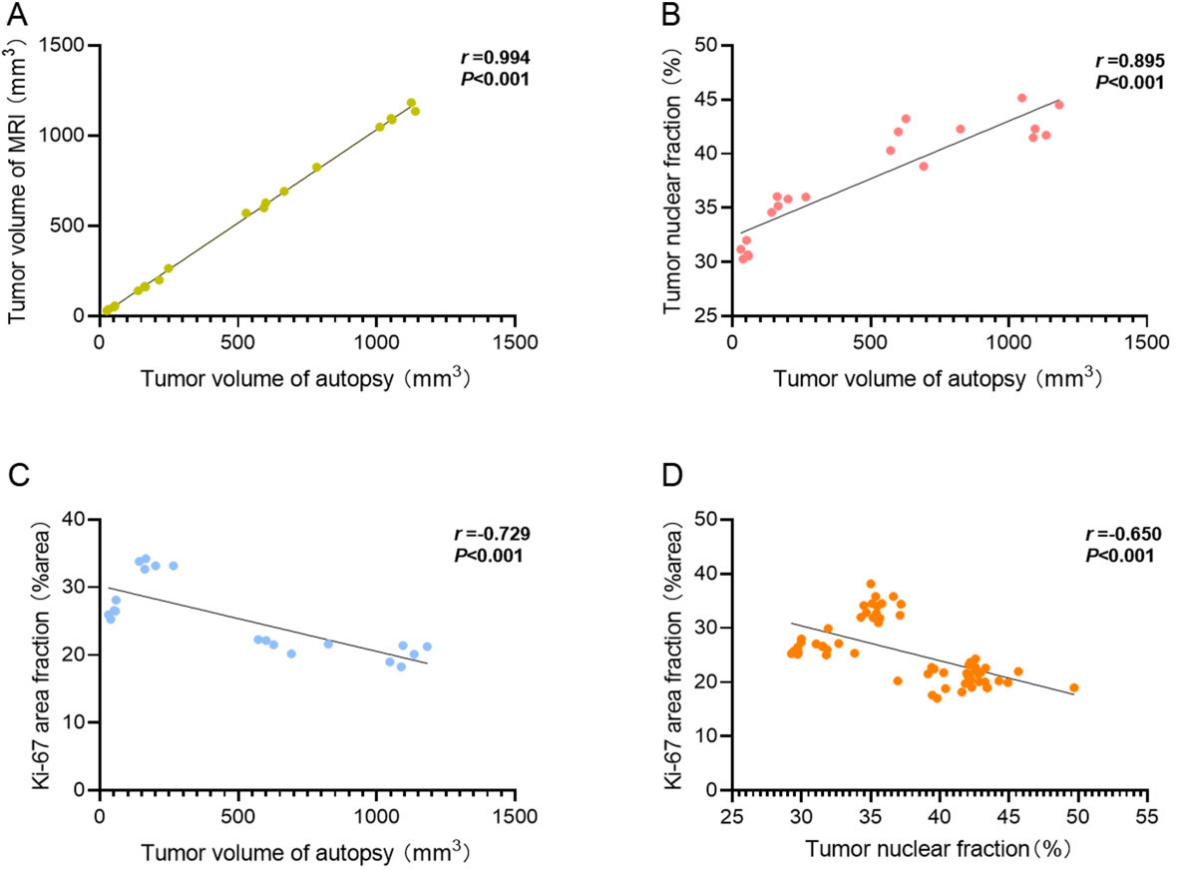
Scatter plot of the correlation analysis of pathological indicators. **A**, Correlation analysis of the experimental group MRI volume and anatomical volume. **B**, Correlation analysis of the experimental group tumor nuclear fraction and anatomical volume. **C**, Correlation analysis of the experimental group Ki-67 area fraction and tumor anatomical volume. **D**, Correlation analysis of the experimental group Ki-67 area fraction and the tumor nuclear fraction. MRI: magnetic resonance imaging.

MRI volume measurements of the mice in the experimental group revealed a strong positive correlation with the volume measured via calipers (r=0.994, P<0.001). The tumor volume was positively and negatively correlated with the tumor nuclear fraction and Ki-67 area fraction, respectively (r=0.895, r=-0.729, P<0.001). The tumor nuclear fraction was negatively correlated with the Ki-67 area fraction (r=-0.650, P<0.001; Figure 7).

The tumor nuclear fraction showed a negative correlation with the ADC values at different b-value combinations, while it had a positive correlation with the EADC values at each b-value combination (Table 5).

**Table 5:** Correlation analysis between MR quantitative parameters and pathological indexes (n=20).

Parameters	b-values	Tumor nuclear fraction		Ki-67 area fraction	
		r	P	r	P
ADC	b=0, 500 s/mm^2^	-0.842	<0.001	0.662	<0.001
	b=0, 1000 s/mm^2^	-0.802	<0.001	0.629	<0.001
	b=0, 1500 s/mm^2^	-0.850	<0.001	0.660	<0.001
	b=0, 2000 s/mm^2^	-0.829	<0.001	0.648	<0.001
	b=500, 1000 s/mm^2^	-0.838	<0.001	0.748	<0.001
	b=500, 1500 s/mm^2^	-0.851	<0.001	0.727	<0.001
	b=500, 2000 s/mm^2^	-0.838	<0.001	0.729	<0.001
	b=1000, 1500 s/mm^2^	-0.859	<0.001	0.647	<0.001
	b=1000, 2000 s/mm^2^	-0.824	<0.001	0.744	<0.001
	b=1500, 2000 s/mm^2^	-0.765	<0.001	0.701	<0.001
EADC	b=0, 500 s/mm^2^	0.884	<0.001	-0.695	<0.001
	b=0, 1000 s/mm^2^	0.847	<0.001	-0.650	<0.001
	b=0, 1500 s/mm^2^	0.846	<0.001	-0.644	<0.001
	b=0, 2000 s/mm^2^	0.807	<0.001	-0.643	<0.001
	b=500, 1000 s/mm^2^	0.823	<0.001	-0.701	<0.001
	b=500, 1500 s/mm^2^	0.781	<0.001	-0.585	<0.001
	b=500, 2000 s/mm^2^	0.677	<0.001	-0.491	<0.001
	b=1000, 1500 s/mm^2^	0.663	<0.001	-0.418	<0.001
	b=1000, 2000 s/mm^2^	0.668	<0.001	-0.503	<0.001
	b=1500, 2000 s/mm^2^	0.562	<0.001	-0.453	<0.001

MR: magnetic resonance; ADC: apparent diffusion coefficient. EADC: exponential apparent diffusion coefficient.

The Ki-67 area fraction was positively correlated with the ADC values at different b-value combinations, while it was negatively correlated with the EADC values at each b-value combination (Table 5).

## Discussion

Investigating prostate cancer requires appropriate animal models. The orthotopic prostate cancer model has a high tumor formation rate and notable repeatability, circumventing the prolonged and inconsistent tumor development time associated with genetically engineered mice. Moreover, this approach maintains the integrity of the mouse immune system. Concurrently, modeling prostate cancer in its native location facilitates accurate replication of the tumor growth and development microenvironment (22), a method that has been extensively employed in related research (23,24). In the present study, C57BL/6J mice were injected *in situ* with a mouse-derived prostate cancer cell suspension (RM-1) to establish a model. The experimental group demonstrated a 100% tumor formation rate with stable tumor growth, providing a solid foundation for subsequent investigations. After tumor implantation, the experimental group initially gained weight due to tumor growth. However, in the later stages, their condition deteriorated progressively, leading to a significant decrease in weight, which was markedly different from the stable weight observed in the control group.

DWI is a noninvasive functional MRI technique that evaluates water molecule diffusion in living tissues and enables the calculation of quantitative parameters such as ADC and EADC values. A brighter DWI signal correlates with lower ADC values, suggesting that a greater degree of water molecule diffusion is limited (12). The diffusion process of water molecules in living tissues is influenced by various structural characteristics of the tissue, including cell density, microvascular perfusion, and the presence of proteins that transport water molecules through the cell membrane. Research indicates that quantitative parameters derived from DWI may offer insights into pathological and biological features, such as cell quantity and tissue microstructure (17,25). These parameters may be effective indicators for evaluating histopathology changes and biological behavior (14-16). However, previous studies did not monitor DWI quantitative parameters under multi b-value combinations during the dynamic process of prostate cancer development (17,18). Therefore, this study was designed to investigate the variations in DWI quantitative parameters across different b-values combinations during tumor progression to identify imaging indices that can accurately reflect changes in tumor histopathology.

In this investigation, DWI of prostate tumors revealed high signals, with the tumor signal intensifying over time. As the b-values increased, the DWI signal progressively diminished, and the signal-to-noise ratio of the image decreased. Nonetheless, the tumor DWI signal remained significantly greater than the surrounding tissues. The signal strength corresponded to the tumor area delineated on T1WI and T2WI, suggesting restricted water molecule diffusion within the tumor region. The ADC values at b-value combinations (b=0, 500/1000/1500/2000 s/mm^2^) in the experimental group were lower than the control group, demonstrating a decreasing trend concurrent with tumor growth. This aligns with the progressive brightening of the DWI signal over time, indicating an escalating restriction of water molecule diffusion in the tumor tissue as the tumor expands. This observation aligns with the findings of Hill et al. (26). In contrast, the EADC values at b-value combinations (b=0, 500/1000/1500/2000 s/mm^2^) for the experimental group exceeded the control group, presenting an upward trajectory with tumor progression that was consistent with DWI signal changes. This trend was inversely related to the changes in the ADC values. The EADC values decreased with increasing b-values at the same scanning time point, mirroring the reduction in the tumor signal visible to the naked eye. These findings suggest that EADC values can be used to quantify the DWI signal and furnish more precise information than visual assessment. This accuracy may be attributed to the method of EADC calculation, which involves dividing the high b-value DWI signal by the low b-value DWI signal to determine the signal intensity ratio of images at two distinct b-values. Since low b-value DWI partially reflects T2WI characteristics, EADC values can mitigate the T2WI projection effect in DWI images, yielding a more accurate DWI signal (27-29).

In addition, we innovated in the combination of b-values and included two nonzero b-values in the mono-exponential model for quantitative parameter calculation. With the exceptions of b=1500, 2000 s/mm^2^, there was no significant difference in the ADC values generated by other b-value combinations between the experimental and control groups on day 9. This finding indicated that these combinations of b-values may be less effective in distinguishing early malignant tissue from normal tissue than the traditional method using b=0 s/mm^2^ for calculating quantitative parameters. Interestingly, unlike the ADC values, the EADC values at b=1500, 2000 s/mm^2^ also faced challenges distinguishing between early tumor and normal prostate tissue, which we attributed to differences in the information represented by the ADC and EADC values. In addition to the fundamental diffusion information of water molecules, the ADC values derived from higher b-value combinations also encompass data on aquaporins that mediate the diffusion and transportation of water molecules across cell membranes. However, the calculation of EADC values is contingent upon tissue signal strength, and further data are needed to ascertain its potential in offering more detailed micro-level information for discriminating between benign and malignant tissues.

For the selection of b-values, this study integrated the range suggested by PI-RADS v2.1 with b-values commonly employed in clinical practice, setting them at 0, 500, 1000, 1500, and 2000 s/mm^2^. These values represent low, medium, and high b-values for evaluating their applicability. Irrespective of the b-value combinations, the ADC and EADC values in the experimental group decreased and increased over time, respectively. The trend of changes across the various b-value combinations was consistent (Figure 4), suggesting that the dynamic alterations in the ADC and EADC values in the prostate cancer mouse model were stable. Moreover, we observed that as the tumor advanced, there was no statistically significant difference in the signal intensity ratio of the tumor at different time points. This finding indicates that the alterations in DWI signal intensity and ADC and EADC values were independent of changes in the T2WI signal, thus demonstrating that the trend of DWI quantitative parameters genuinely and effectively reflected the alteration of water molecule diffusion and microstructure in tumor tissues. In the control group, the repeated scanning results revealed that the ADC and EADC values of the different b-value combinations did not change with time. This stability might serve as an effective metric for the longitudinal assessment of prostate cancer onset and progression. Nonetheless, there was no marked difference in the ADC and EADC values at some adjacent time points in the experimental group, mirroring findings by Hectors et al. (25), who reported insignificant ADC value variances among patients with differing Gleason scores and between the pT2 and pT3 stages. This could be attributed to the influence of the tumor microenvironment on water molecule diffusion, where factors such as the presence of collagen, matrix swelling, and stromal disintegration within the tumor might impact the quantitative DWI parameters. Alternatively, the lack of significant differences could result from the study's limited sample size and brief data collection interval. These aspects will be addressed and improved upon in future research endeavors.

In the experimental group, prostate tumor cells rapidly proliferated, compromising the integrity of basal cells and invading normal glandular structures. This behavior aligns with hallmark features of prostate cancer, such as the degeneration of normal gland architecture, heightened tumor cell density, and irregular distribution of these cells (4). Typically, tumors at more advanced stages are characterized by greater cell density (17). Consequently, this study employed the tumor nuclear fraction observed via HE staining as a proxy for tumor cell density, which indirectly reflects alterations in tumor cell quantity. The findings revealed that the tumor nuclear fraction increased as the tumor progressed, mirroring the tumor's aberrant proliferation characteristic. Between days 15 and 18, no significant variance was observed in the tumor nuclear fraction, potentially attributable to the swift expansion of tumors in early stages compared with the decelerated growth in later stages. The occurrence of hemorrhage, necrosis, vacuolation, and hyalinization within the tumors may also contribute to the decrease in cell quantity observed (30). In addition, the dynamic expression of Ki-67 was detected by immunohistochemistry to reflect tumor proliferation. The results revealed that the tumors had proliferation ability at all time points. Quantitative analysis showed that the Ki-67 area fraction decreased on day 15 indicating that the proliferation ability of tumor cells decreased. This finding also explains why the tumor nuclear fraction measured by HE staining did not increase significantly between days 15 and 18.

Correlation analysis revealed a strong positive relationship between tumor volume determined through anatomical measurements and MRI within the experimental group. This finding underscores the efficacy of MRI as a noninvasive, accurate tool for monitoring tumor growth and evaluating treatment efficacy, which aligns with the findings of Ni et al. (31). Additionally, a positive correlation was observed between tumor nuclear fraction and tumor volume assessed by anatomical measurements, suggesting that MRI-based volume measurements not only precisely reflect changes in tumor size but also may infer shifts in tumor cell quantity. A negative correlation was detected between the tumor nuclear fraction and the ADC values across all b-value combinations, implying that tumor cell proliferation constricts the interstitial and intraluminal spaces, thereby restricting water molecule diffusion (32,33). Conversely, a positive relationship was found between the tumor nuclear fraction and the EADC values at all b-value combinations, indicating the ability of the EADC to accurately quantify the DWI signal intensity and, in conjunction with the ADC values, delineate water molecule diffusion. The tumor nuclear fraction significantly increased from days 9 to 15, followed by a minor increase from days 15 to 18, mirroring the decreasing trend of the ADC over the same period. These results suggest a biophysical relationship between the ADC and EADC values and the pathological structure of prostate cancer. In this investigation, the ADC values at b=1000, 1500 s/mm^2^, as well as the EADC values at b=0, 500 s/mm^2^, demonstrated the strongest correlations with the tumor nuclear fraction (r=-0.859 and 0.884, P<0.001, respectively). The correlation between the ADC values and the tumor nuclear fraction consistently remained close to -0.800 across all b-value combinations, indicating the robustness of the ADC values in indirectly reflecting cell density. However, for combinations of b-values other than 0 s/mm^2^, the correlation between EADC values and the tumor nuclear fraction gradually decreased with increasing adopted b-values and reached its minimum at b=1500, 2000 s/mm^2^ (r=0.562). This finding diverges from previous studies (34-36) that showed that high b-values possess greater application value, potentially due to variations in research subjects, equipment, and evaluative indices.

In addition, the results revealed that Ki-67 expression was negatively correlated with tumor volume and nuclear fraction, positively correlated with the ADC value at each b-value combination, and negatively correlated with the EADC value. This may be due to the gradual increase in the density of tumor cells and the increased restriction of the water molecule diffusion in the tumor with progression. As a result, the proliferation ability of tumor cells, which urgently need water, blood, and nutrients to maintain growth, is also limited; therefore, the proliferation ability of tumor cells is reduced. In this study, the ADC and EADC values at b=500, 1000 s/mm^2^ were more strongly correlated with Ki-67 than the other b-value combinations (r=0.748, -0.701, P<0.001), and the ability of DWI-based quantitative parameters to characterize cell proliferation was less intuitive than that of cell density. In general, there is a close correlation between tumor nuclear fraction, Ki-67 expression, and DWI quantitative parameters, which has not been discussed in other studies and needs further exploration.

In conclusion, the longitudinal assessment of ADC and EADC values facilitated tumor progression monitoring, presenting a potential alternative imaging biomarker for quantifying the quantity and proliferation ability of prostate cancer cells. The ADC values at b=1000, 1500 s/mm^2^ and the EADC values at b=0, 500 s/mm^2^ may be most valuable for evaluating cell quantity. The ADC and EADC values at b=500, 1000 s/mm^2^ may be most valuable for evaluating tumor proliferation ability. Future research should explore the relationships between the quantitative parameters of DWI sequences and the characteristics of luminal spaces and more pathological indicators to investigate the potential of MRI for the non-invasive estimation of pathological alterations in prostate cancer tissues.

## Supplementary Material

Click to view [pdf].
