# Supplementary material for: The dynamic changes in quantitative parameters of diffusion-weighted imaging at different b-values in a prostate cancer mouse model and their correlation with histopathology parameters
Source: Braz J Med Biol Res. 2025 Aug 29;58:e14527. doi: 10.1590/1414-431X2025e14527 (PMC12396619; doi:10.1590/1414-431X2025e14527)
Supplement: Supplementary file 1 [file 1414-431X-bjmbr-58-e14527-suppl.pdf]

Table S1. Comparison of apparent diffusion coefficient (ADC) values ($\times10^{-3}$ mm^2/s) from b-value combinations (b=500, 1000/1500/2000 s/mm^2), (b=1000, 1500/2000 s/mm^2), and (b=1500, 2000 s/mm^2) in each group (n=5).

Group	b=500, 1000 s/mm^2		b=500, 1500 s/mm^2		b=500, 2000 s/mm^2		b=1000, 1500s/mm^2		b=1000, 2000 s/mm^2		b=1500, 2000 s/mm^2	
	Model	Control	Model	Control	Model	Control	Model	Control	Model	Control	Model	Control
Day 9	0.9 ± 0.13^{a}	0.89 ± 0.05^{a}	0.81 ± 0.13^{a}	0.8 ± 0.05^{a}	0.69 ± 0.1^{a}	0.7 ± 0.05^{a}	0.7 ± 0.15^{a}	0.72 ± 0.06^{a}	0.58 ± 0.11^{a}	0.63 ± 0.07^{a}	0.48 ± 0.08^{a}	0.52 ± 0.07
Day 12	0.86 ± 0.08^{a}	0.89 ± 0.04^{a}	0.74 ± 0.11^{a}	0.8 ± 0.05	0.65 ± 0.08^{a}	0.68 ± 0.04^{a}	0.54 ± 0.08^{b}	0.71 ± 0.03	0.53 ± 0.09^{a}	0.61 ± 0.03	0.46 ± 0.11^{a}	0.51 ± 0.03
Day 15	0.47 ± 0.08^{b}	0.88 ± 0.05	0.43 ± 0.07^{b}	0.79 ± 0.05	0.38 ± 0.06^{b}	0.72 ± 0.06	0.34 ± 0.07^{c}	0.74 ± 0.05	0.31 ± 0.06^{b}	0.63 ± 0.06	0.3 ± 0.08^{b}	0.52 ± 0.06
Day 18	0.34 ± 0.03^{c}	0.87 ± 0.05	0.33 ± 0.03^{b}	0.78 ± 0.05	0.33 ± 0.04^{b}	0.69 ± 0.04	0.31 ± 0.07^{c}	0.74 ± 0.03	0.29 ± 0.05^{b}	0.63 ± 0.06	0.25 ± 0.03^{b}	0.54 ± 0.06
F/H	148.2	1.702	48.54	0.9894	46.32	2.192	53.46	9.353	51.66	2.553	41.67	0.8219
P	<0.001	0.6366	<0.001	0.4045	<0.001	0.5336	<0.001	0.0249	<0.001	0.4657	<0.001	0.4873

Data are reported as means and SD. Different letters indicate statistically significant differences ($P<0.05$; ANOVA and Kruskal-Wallis test). F/H: statistical results of data comparison among multiple groups.

Table S2. Comparison of exponential apparent diffusion coefficient (EADC) values from b-value combinations (b=500, 1000/1500/2000 s/mm^2), (b=1000, 1500/2000 s/mm^2), and (b=1500, 2000 s/mm^2) in each group (n=5).

Group	b=500, 1000 s/mm^2		b=500, 1500 s/mm^2		b=500, 2000 s/mm^2		b=1000, 1500s/mm^2		b=1000, 2000 s/mm^2		b=1500, 2000 s/mm^2	
	Model	Control	Model	Control	Model	Control	Model	Control	Model	Control	Model	Control
Day 9	0.5 ± 0.07^{d}	0.42 ± 0.04	0.39 ± 0.04^{d}	0.33 ± 0.03	0.34 ± 0.04^{c}	0.29 ± 0.03	0.39 ± 0.05^{b}	0.33 ± 0.04	0.36 ± 0.06^{c}	0.3 ± 0.03	0.37 ± 0.06^{c}	0.34 ± 0.03^{c}
Day 12	0.64 ± 0.07^{c}	0.43 ± 0.03	0.58 ± 0.07^{c}	0.35 ± 0.03	0.51 ± 0.04^{b}	0.31 ± 0.03	0.58 ± 0.16^{a}	0.35 ± 0.02	0.47 ± 0.11^{bc}	0.31 ± 0.04	0.43 ± 0.08^{bc}	0.35 ± 0.03
Day 15	0.72 ± 0.03^{b}	0.42 ± 0.03	0.65 ± 0.04^{b}	0.34 ± 0.03	0.54 ± 0.06^{b}	0.3 ± 0.02	0.65 ± 0.11^{a}	0.35 ± 0.02	0.54 ± 0.09^{ab}	0.32 ± 0.03	0.48 ± 0.07^{ab}	0.36 ± 0.03
Day 18	0.86 ± 0.06^{a}	0.43 ± 0.03	0.74 ± 0.08^{a}	0.35 ± 0.03	0.67 ± 0.12^{a}	0.29 ± 0.04	0.68 ± 0.11^{a}	0.34 ± 0.02	0.59 ± 0.1^{a}	0.3 ± 0.03	0.54 ± 0.08^{a}	0.33 ± 0.02
F/H	92.21	1.077	90.18	2.37	56.38	1.912	34.05	1.495	31.14	0.6393	25.48	5.29
P	<0.001	0.3662	<0.001	0.0802	<0.001	0.1381	<0.001	0.2307	<0.001	0.5929	<0.001	0.1517

Data are reported as means and SD. Different letters indicate statistically significant differences ($P<0.05$; ANOVA and Kruskal-Wallis test). F/H: statistical results of data comparison among multiple groups.
